# Cyclization of Short Peptides Designed from Late Embryogenesis Abundant Protein to Improve Stability and Functionality

**DOI:** 10.1002/cbic.202401013

**Published:** 2025-02-20

**Authors:** Yinghan Wu, Shinya Ikeno

**Affiliations:** ^1^ Department of Biological Functions Engineering Kyushu Institute of Technology Kitakyushu Science and Research Park Kitakyushu Fukuoka Japan

**Keywords:** LEA peptide, cyclization, SICLOPPS, drought stress

## Abstract

LEA peptides, which are designed based on late embryogenic abundant (LEA) protein sequences, have demonstrated chaperone‐like functions, such as improving drought stress tolerance of Escherichia coli (*E. coli*). Previous studies have focused on the biological functions of linear LEA peptides. However, the function of cyclic LEA peptide still unknown. This study aimed to explore the cyclic LEA peptides’ bio function like enhance the drought stress tolerance of *E. coli* by cyclizing the LEA peptide using SICLOPPS (Split Intein Circular Ligation of Peptides and Proteins). The results indicated that cyclization significantly improved the function and extended the potential applications. At the same time, we found that peptides containing numerous lysine residues exhibited reduced performance, which may be due to the exteins’ residues affecting the SICLOPPS efficiency.

## Graphical abstract

Late embryogenic abundant (LEA) peptides are short, disordered peptides that enhance *Escherichia coli*′s drought stress tolerance. Cyclization of LEA peptides can further improve their properties, such as anti‐desiccation, thereby expanding their potential applications

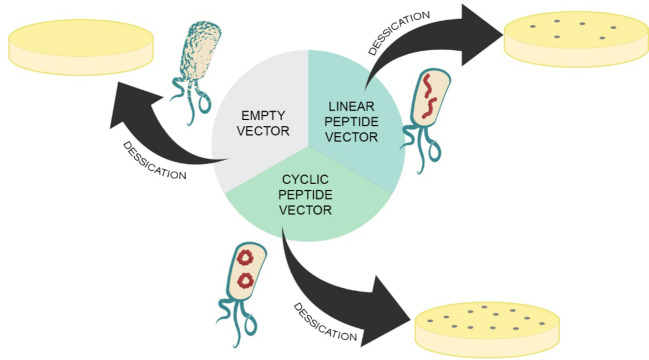

## Introduction

Late embryogenesis abundant (LEA) proteins families widely exist in maturing seeds, anhydrobiotic plants, animals, and microorganisms, usually accumulated in the organisms’ bodies in response to the environmental desiccation stress tolerance.^[1]^ LEA proteins have a chaperone‐like function, and their upregulation is associated with various abiotic stresses, including desiccation, osmotic stress, high salinity, and extreme temperature.^[2]^ Those chaperon‐like LEA proteins are mainly classified as the Group 3 LEA proteins (G3LEA), a family of proteins characterized by their repeating and conserved tandem 11‐mer motifs. For example, Hatanaka et al. observed that a considerable number of PvLEA4 proteins (kind of G3LEA protein) accumulated in the almost completely dehydrated larvae‘s body.^[3]^ Furuki et al. discovered that the G3LEA protein model peptides also can prevent the aggregation of client proteins, which they referred to as molecular shield activity.^[4]^ In their experiment, a 22‐mer peptide was chemically synthesized and can prevent the aggregation of the lysozyme under heat‐induced stress and maintain the lysozyme's bioactivity. In our lab, we primarily focus on the 11‐mer unit's function. A variety of 11‐mer LEA‐like peptides were designed based on the 11‐mer conserved unit (ex. LEA S, LEA II, LEA K, and LEA E).[^5^, ^6^] These LEA peptides were produced by intracellular synthesis to directly analyze their biofunctions in the cells. Relative anti‐abiotic stress tests have been performed to analyze their functions in the cell and those studies have indicated that 11‐mer LEA peptides exhibit chaperone‐like activity *in vivo*.[^6^, ^7^, ^8^] Until now, LEA peptides have been shown to enhance bacterial tolerance against abiotic stresses including UV,^[6]^ salt, heat, cold,^[7]^ and acid.^[8]^

However, the bio‐functions of LEA peptides were studied just in linear form, and their cyclic form still unknown. The benefit of the cyclic structure is its resistance to hydrolysis by exopeptidases due to the lack of both amino and carboxyl termini. The rigidity of cyclic peptides decreases the entropy term of the Gibbs free energy, thereby enhancing binding toward target molecules or receptor selectivity.^[9]^

In this study, we investigated the bioactivity of LEA peptides whether can be enhanced by cyclization, such as improve the cell's desiccation tolerance comparing the linear and cyclic form.

**Figure 1:**
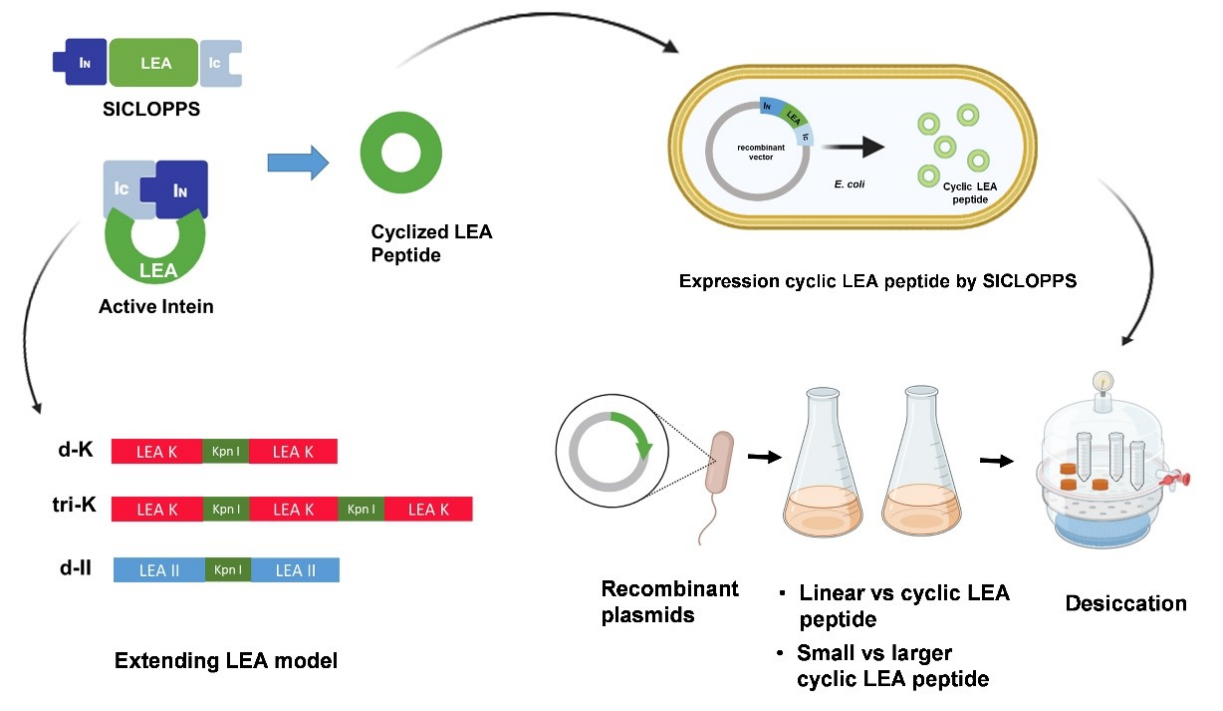
Cyclizing LEA peptides by SICLOPPS. The linear and cyclic LEA peptides’ capacity to improve cell desiccation tolerance was comparatively analysed. Large cyclic LEA peptides were also produced by extending the LEA model to compare the small and large cyclic LEA peptides′ functions.

LEA II (MDAKDGLKEKAGE) and LEA K (MDAKDKLKEKAKE) were selected, because LEA K shown highest activity in previous studies and LEA II is physiology neutral.[^7^, ^8^] SICLOPPS was used to cyclization the peptides’ chain and Escherichia coli (*E. coli*) was selected as the model organism. As shown in Figure 1, the LEA peptides were presented in the extein position, which looks like I_N_‐LEA peptide‐I_C_, and the N‐ and C‐terminals of the LEA peptide chain can be ligated as head‐to‐tail cyclization *via* SICLOPPS. The linear LEA II and LEA K also subcloned into the same restriction enzyme cut site as contrast study. First, we compare the cyclic versus linear LEA peptides to assess their impact on the desiccation tolerance of *E. coli*. The quantitative polymerase chain reaction (qPCR) was employed to analyze the expression levels of interest genes. The computational simulation of inteins splitting and ligation process was performed using Molecular Operating Environment (MOE 2018) software. Furthermore, we investigate peptides with longer chain lengths that are extended by repeating LEA peptide units, to enhance stress resistance functions.

## Results and Discussion

**Figure 2:**
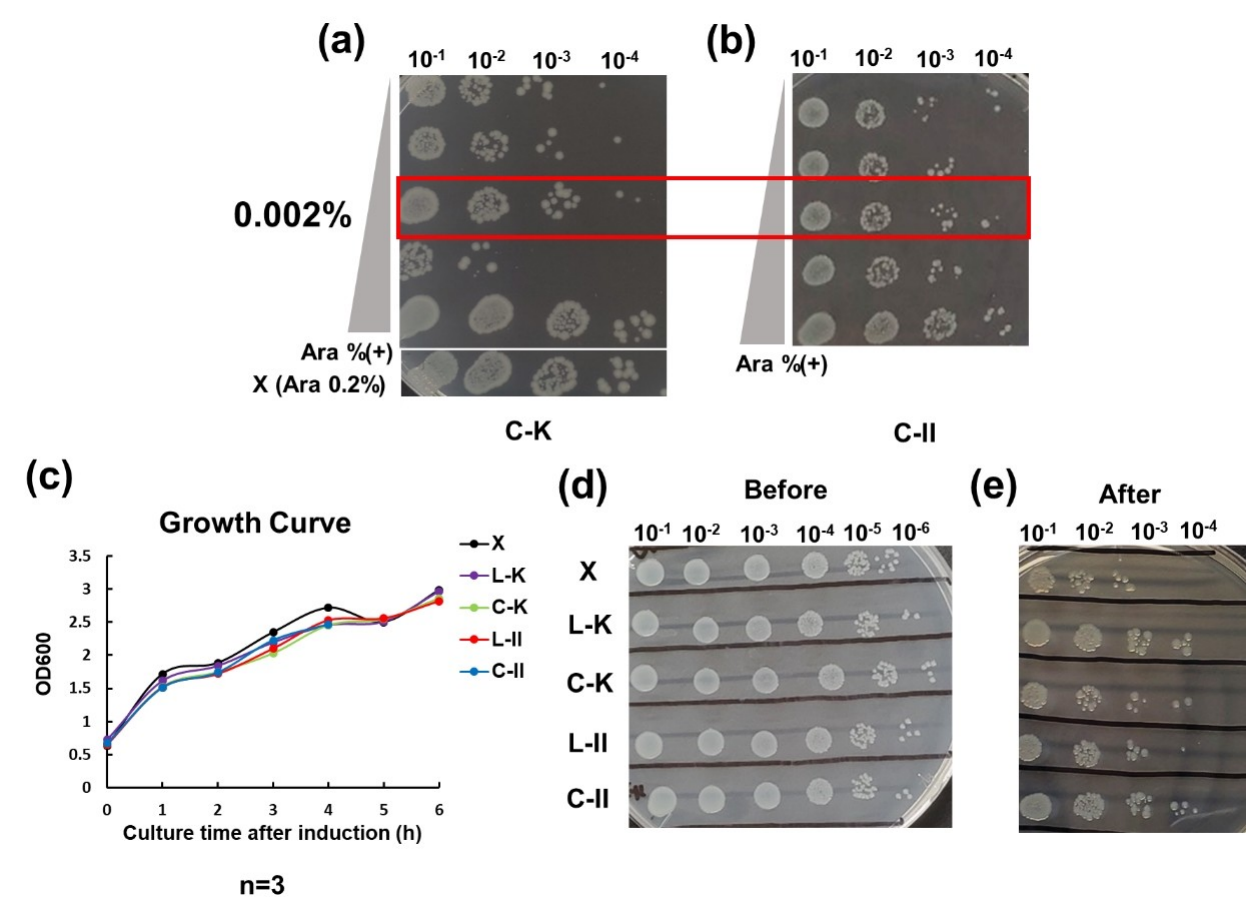
Desiccation resistance test results. (a) Drop spotting analysis of C−K desiccation resistance under different Ara concentrations; X is the control sample, transformed with empty pBAD and induced by 0.2 % Ara. (b) Drop spotting analysis of C‐II desiccation resistance under different Ara concentrations. (c) Growth curve of X, L−K, C−K, L‐II, and C‐II under 0.002 % Ara induction (n=3). (d) and (e) Drop spotting images of samples before and after desiccation when induced by 0.002 % Ara.

The inducer concentration was determined by the number of surviving colonies of *E. coli* after desiccation at different induced arabinose (Ara) concentrations (0.0, 0.001, 0.002, 0.02, and 0.2 %). As Figure 2a, b suggested, the 0.002 % concentration of Ara was selected and used in the following analysis as the bacteria exhibited certain desiccation tolerance under this inducer concentration. The survival colony number decreased when induced with 0.02 % Ara. This insight suggests that the overexpression of SICLOPPS’ inteins brought certain metabolic stress and affected cell survival ability. At the highest Ara concentration, we observed the phenomenon of false positives happened. Figure 2a demonstrates that the control (X) also exhibited a certain degree of desiccation tolerance when induced by 0.2 % Ara. It is well‐known that sugar, such as Ara, is a potent desiccation protector and carbon source see reference.^[10]^ This may explain the high survival colony number observed. However, the highest inducer concentration may not truly reflect the peptides′ ability to enhance cell desiccation tolerance. As the experiment‘s purpose is to investigate the cyclic LEA peptides′ function, we attempted to limit the negative effect of overexpression when utilizing the SICLOPPS. We found that *E. coli* had similar growth rates under 0.002 % Ara induction (Figure 2c), and the colony numbers were not significantly different from each other (Figure 2d). At the same time, samples shown significant differences before and after dissociation (Figure 2d, e). As a result, the 0.002 % Ara concentration was deemed an optimal inducer concentration for SICLOPPS expression in *E. coli*, as it effectively mitigates the metabolic effect and avoids false positives.

**Figure 3:**
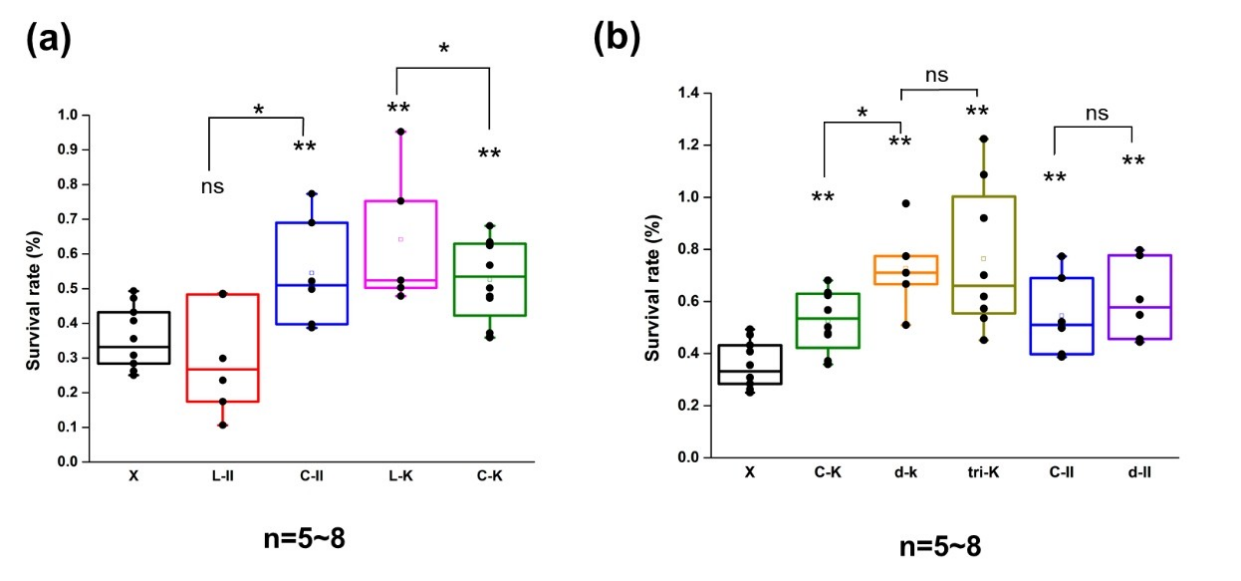
Results of desiccation resistance test. (a) The desiccation survival rate of linear versus cyclic LEA peptide samples. Data are mean±SD (n=5~8). (b) The desiccation survival rate of larger cyclic LEA peptide samples. Data are mean±SD (n=5). *, significance at P <0.05; **, significance at P<0.01; ns, not significant.

The desiccation survival ratio of *E. coli* was determined by CFU counting. The majority of cells are unable to be cultivated and repopulated even after rehydration due to the destructive effects of dehydration on cellular protein structure, DNA damage, and oxidative stress.^[11]^ As Figure 3a can be seen, the cells’ population dramatically decreased after 6 h desiccation, with the control group (X) exhibiting a mere 0.3 % survival rate. The survival rate of L‐II was close to X and without statistically significant difference. It appears that the expression of LEA II does not enhance bacterial dissociation tolerance. Notably, the survival ratio of C‐II was approximately 1.6 times higher than that of the control. In the case of LEA K, L−K exhibited the most robust desiccation tolerance, with a survival ratio that improved by approximately 1.9‐fold. The remarkable protective capacity of LEA K is consistent with our previous findings, which demonstrated that LEA K exhibited the most robust protective capacity under diverse abiotic stressors (e. g., salt, UV, pH).^[8]^ Regrettably, C−K was unable to enhance the efficacy or functionality of LEA K. It exhibited a certain degree of desiccation tolerance but was not as pronounced as that observed in L−K and X.

To gain a better understanding of the differences between LEA II and LEA K, we conducted qPCR to analyze the relative expression levels of LEA II and LEA K. Molecular dynamics (MD) simulations were performed to mimic the splitting and cyclization process of LEA II and LEA K. According to the analyzed results (see below), the larger‐size cyclic LEA peptides were studied. An investigation strategy was carried out to enhance the flexibility of the peptide chain by extending the repeat LEA motif (Figure 1).

As Figure 3b shows, the performance of cyclic LEA K was enhanced by extending the LEA K repeat. The marginal gain was primarily observed when extending one repeat, d‐K and tri‐K display similar survival rates. In addition, the dissociation results of C‐II and d‐II exhibited similar survival rates and no statistical significance difference. A research group led by Bob and Jonathan has demonstrated that the minimal LEA motif is sufficient to enhance desiccation tolerance.^[12]^ It is hypothesized that the improvement observed in d‐K is due to the longer peptide chain improving the poor ligation efficiency.

The expression levels of LEA II and LEA K were determined by a two‐step qPCR including cyclic and linear samples. The expression level of LEA K was employed as the control, and 16S rRNA was utilized as the endogenous control, as shown in Table 1. The relative expression levels of L‐II and C‐II are both higher than those of L−K and C−K. In particular, L‐II was higher than L−K by 2.16 folds, while C‐II was higher than C−K by 4.4 folds. This suggests that there is no significant positive correlation between the expression level of the LEA gene and the enhancement effect toward desiccation tolerance. L−K exhibited the most robust protective effect, yet its expression level was not significantly higher than that of L‐II or C‐II. Nevertheless, we hypothesize that the true expression level of C−K may be lower than that of the exception.

**Table 1:**
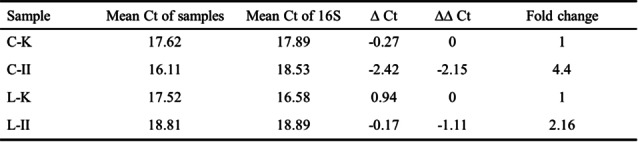
qPCR analysis of linear and cyclic LEA peptides.

**Figure 4:**
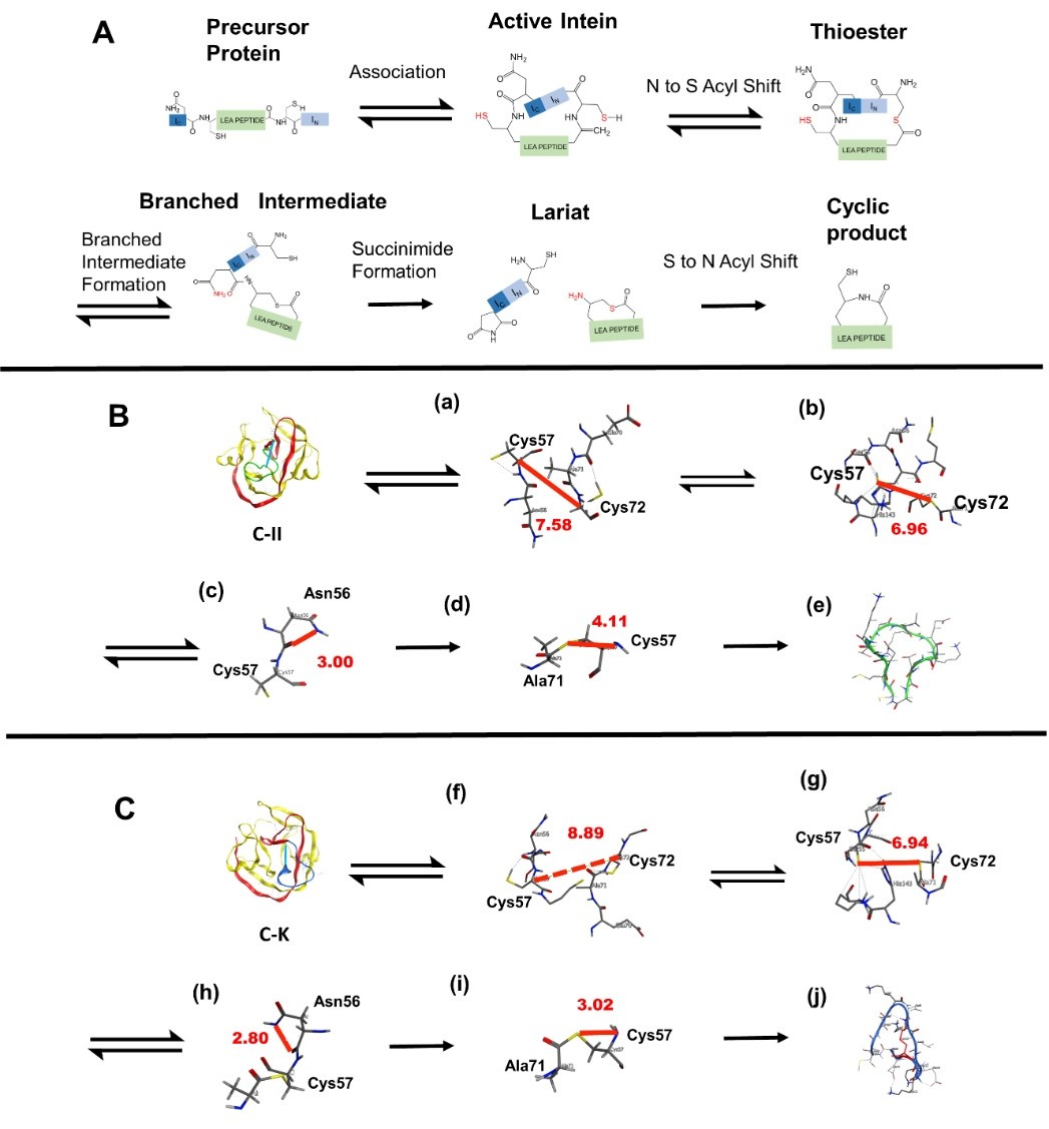
SICLOPPS cleavage and cyclization processes. A schematic representation of SICLOPPS’ splitting and cyclization mechanism. B the predictive structure of the C‐II precursor, the split‐intein is dawn as ribbon (IN: yellow; IC: red) and the C‐II peptide is green. C, the predictive structure of the C−K precursor, the split‐intein is dawn as ribbon (IN: yellow; IC: red) and the C−K peptide is blue. (a, b, c, d) The side view of active groups of the C‐II splitting and ligation process, the relative active groups are shown in red letters on the above scheme. (f, g, h, i) The side view of active groups of C−K splitting and ligation process, the presenting order same as C‐II. (e) The structure of cyclic LEA II. (j) The structure of cyclic LEA K. All screenshots are 0 ns of conformations’ molecular dynamic simulation; red solid line, the distance of active groups, red dot line, strong interaction.

The MOE software was employed to run the MD simulation of C‐II and C−K, respectively. The classical splicing process of inteins is comprised of four transfer reaction steps (Figure 4A): 1) N to S acyl shift, 2) branched intermediate formation, 3) succinimide formation, and 4) S to N acyl shift.^[13]^ The conformations of four transfer reaction steps were analyzed step by step at 500 ns MD in MOE. Individual ligation steps were then manually performed in order. The initial mimic models (Figure 4B, C) predicted from SWISS‐MODEL showed tropical horseshoe‐like beta‐sheet folds consistent with the findings of Shah^[14]^ and Liu.^[12]^ Before the N to S shift, two active splicing sites (Cys57 and Cys72) of C‐II and C−K were initiated with comparable distances at 7~9 Å (Figure 4a, f). Furthermore, a robust interaction between the thiol group of Cys72 and the carbonyl group of Ala71 in the active inteins conformation of C−K (Figure 4 f) was observed. This conformation facility allows for the N to S acyl transfer reaction and insight into the C−K conformation may result in the splitting process being initiated preferentially. As the stimulation proceeded, the distance between the two active sites gradually closer to approximately 3 Å (Figure 4a to c; Figure 4 f to h). Until this step, C‐II and C−K showed a similar conformation‐changing process.

**Figure 5:**
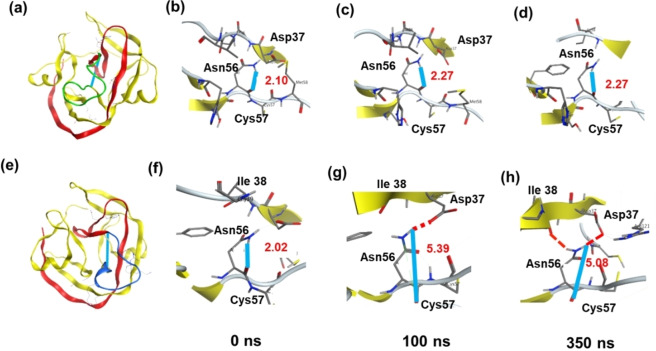
Molecular dynamic simulation of the branched intermediate step. (a) Overview of C‐II structure. (b), (c), and (d) are the site views of C‐II conformations at 0, 100, and 350 ns, respectively. (e) Overview of C‐K structure. (f), (g), and (h) are the site views of C‐K conformations at 0, 100, and 350 ns, respectively. Blue solid line, the distance of active groups; red dot line, strong interaction.

The branched intermediate formation is characterized by nucleophilic attack of the side‐chain primary amide group of Asn residue on the adjacent peptide bond.^[15]^ This process is illustrated in Figure 4c, h, which depicts the N−H‐O hydrogen bond coupling the Asn56 side chain and the carbonyl group of Cys at 0 ns with a distance of ~2 Å. This bond is present in both the C‐II and C−K conformations. In the C‐II case, only one conformation was observed during the simulation process. The two key side chain groups exhibited a strong interaction and maintained a close distance throughout (Figure 5b to d). This suggests that the next step, the succinimide formation, is more likely to occur. In contrast, at C−K, the distance between the amino group of Asn56 and the carbonyl group of the backbone was observed to be increasing (Figure 5f to h). At 100 ns, the Asn56 side chain adopts the carbonyl group of Asp37. Subsequently, the side chain of Asn56 was attracted by another carbonyl group of Ile38 and it ultimately interacts with both Asp37 and Ile38, exhibiting distinct conformations in comparison to those observed with C‐II. The N to S acyl shift and branched intermediate formation process are reversible reactions, with the rate‐limiting step being the succinimide formation process.[^16^, ^17^] This may provide insight into the reason why C‐II and C−K have different performances.

Peptide forms the lariat conformation after succinimide formation. At this step, the free amino group of Cys57 attacks the thioester group of Ala71. As Figure 4d and Figure 4i illustrate, the two reaction groups were C−K closer than C‐II. Finally, the S to N shift produces a peptide bond and the cyclic LEA peptides (Figure 4e, j).

These findings indicate that containing numerous lysine residues affects the SICLOPPS efficiency and resulting poor cyclic product.

In summary, the initial phase of the study involved a comparison of linear and cyclic LEA peptides. Subsequently, the effects of larger cyclic LEA peptides were also examined. It was postulated that the cyclization of LEA could enhance the functionality of linear LEA peptides. In the linear and cyclic comparison analysis, linear LEA K exhibited the strongest resistance to desiccation, while the difference between L‐II and the control group was marginal. Cyclization was found to be an effective method of improving the functionality of linear LEA II, although C−K did not demonstrate superior performance to L−K. qPCR analysis revealed that the expression level of LEA K was lower than that of LEA II, both in linear and cyclic forms. However, C−K exhibited a much lower expression level than L−K, likely due to the relatively low yield of cyclic LEA K. The MD simulation of C‐II and C−K indicated that the structural conformation of C−K was more prone to initiate cleavage and subsequent cyclization, however, the whole process of splitting and ligation was slower. Because at the rate‐limiting step, intein‐succinimide formation occurs more frequently in C‐II than in C−K. This also provides insight into the relatively low yield of cyclic LEA K. In larger‐size cyclic LEA studies, the performance of cyclic LEA K improved by extending minimal LEA motifs. However, the longer LEA peptide chain does not result in a better marginal benefit. Similarly, in LEA II, d‐II and C‐II show approximate survival rates as extending LEA‐II doesn't improve the cyclic LEA II performance. This is evidenced by a peer study that demonstrated the efficacy of a minimal LEA motif in mitigating desiccation stress.^[18]^

## Conclusions

In conclusion, this study represents an inaugural investigation into the influence of cyclization on the functionality of chaperone‐like peptides *in vivo*. The results of this study are intriguing, as they demonstrate that cyclization confers certain benefits to the linear counterpart, including enhanced peptide stability. Nevertheless, it is essential to consider certain factors that may influence the outcome of future studies. These include the additional metabolic consumption associated with bacterial expression of inteins and the potential for low target yield, which require further investigation.

## Experimental Section

**Plasmids Construction**. We followed the Townend et al. method with slight modifications.^[19]^ The gene of SICLOPPS inteins encoding with LEA II (pBAD‐SICLOPPS‐LEA II) was synthesized de novo by Eurofins. The pBAD‐SICLOPPS‐LEA II was used as the DNA template and carried out point mutation to obtain cyclic LEA K recombinant plasmid (pBAD‐SICLOPPS‐LEA K). Two pairs of primers, G6K and G12K, were used in this experiment. The sequences can be found in Sup. Table 1.

Comparatively, the linear LEA II (L‐II) and linear LEA K (L−K) were also subcloned into pBAD at the *Nco*I and *Hind*III sites. The oligo DNA pairs, *Nco*I‐LEA K‐*Hind*III and *Nco*I‐LEA II‐*Hind*III (Sup. Table 1) were purchased from the company and subclone to the expression vector. Briefly, the purchased oligo DNA segments and expression vector pBAD (Novagen) were treated with *Nco*I and *Hind*III restriction enzymes and cleaned up by the PCR Clean‐Up kit (QIAGEN, USA). The purified inserts and vector genes were ligated with Ligation Mix (Takara, Japan) and transformed to *E. coli* nova blue, respectively.

The detailed amino acid sequence of SICLOPPS cyclic LEA II used in this study is given below, the different portions of LEA K amino acid reside highlighted with gray, and the SsrA sequence: NFALKNGFIASNC is in BOX:

MHHHHHHGENLYFKLQAMGMIKIATRKYLGKQNVYDIGVERYHNFALKNGFIASNC **MDAKDGLKEKAGE** ACLSYDTEILTVEYGILPIGKIVEKRIECTVYSVDNNGNIYTQPVAQWHDRGEQEVFEYCLEDGCLIRATKDHKFMTVDGQMMPIDEIFERELDLMRVDNLPNGTAANDENYALAA.

To construct the two repeat LEA motifs in SICLOPPS using the In‐fusion kit (Takara, Japan). The vectors were amplified by Infusion dK/Infusion dII primer pairs, respectively. While the oligo insert pairs were purchased, including Insert K‐1; Insert K‐2; Insert II‐1; and Insert II‐2. The Kpn I restriction cutting site also be introduced at the same time (see Fig. 1) which allows the third LEA K directly inserted at the Kpn I site based on the d‐K reconstructed plasmids. For sequences see Sup. Table 1.

**Cell Culture** *E. coli* BL21 (DE3) were transformed with diversity reconstruction plasmids by heat shock. The bacterial culture process followed the previous method,^[8]^ briefly, all transformed *E. coli* were spread on the Luria‐Bertani (LB) plates supplemented with 100 μg/mL ampicillin, and then incubated overnight at 37 °C. The single colony was used to inoculate 3 mL of LB liquid medium overnight for pre‐culture. The culture was diluted 100‐fold with fresh LB (Amp+) and incubated until the optical density OD_600_ reached ~0.6, induced with arabinose (Ara), and incubated for an additional 4 hours. Cell growth was detected at an optical density of 600 nm. Except for special mention, the same culture medium was used in this study.

**Desiccation** The pellet used for desiccation is equivalent to a 25 mL incubation medium. The 25 mL harvested incubation sample mediums were centrifuged for 15 min at 4,000 g, and the supernatants were discarded. The pellets were resuspended in 5 mL of phosphate‐buffered saline (PBS) to achieve the cells around 1.1×10^9^ cells/mL. The colony‐forming units (CFU) were counted with series dilution, and the CFU value was estimated as the initial E. coli survival number (*N_i_*) without treatment. Centrifuge for another 5 min at 4,000 g to remove the supernatants, then all sample pellets were desiccated together for 6 hours in the desiccator at 25 °C, 20~40 % humidity. After complete drying (~6 hours), the bacterial pellets were rehydrated with 5 mL PBS, and their CFU numbers were counted as *E. coli* survival numbers (*N_v_*). The survival ratio of bacterial calculated with the formula:

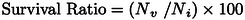




**Molecular Dynamic Simulation** Initial protein structures for the molecular dynamic (MD) simulations were predicted on the SWISS‐MODEL website.^[20]^ The model with the highest score, ranked by QMEANDisCo Global, was selected to simulate of SICLOPPS inteins ligation process. The simulation process was presented by MOE software (vs. 2018). The monomer structure of predicted SICLOPPS (C‐II and C−K) models were prepared for simulation analysis, respectively, containing a series of undesired chain deletion, energy minimization, solvent, and force field defaults (AMEBER 10). The NPA algorithmic with 500 ns simulation was performed to analyze the conformations and manually mimic the inteins ligation process.

**qPCR analysis** The expression of L‐II, C‐II, L−K, and C−K genes is relatively quantified by two‐step qPCR. The total RNA of L‐II, L−K, C‐II, and C−K were extracted after inducement with 0.002 % Ara and 4 h culture. RNeasy Mini column (Qiagen, USA) kit was used to extract and purify the total RNA, the operation following the manufacturer's instructions. Around 1 μg sample of total RNA was used for cDNA synthesis, and then reverse transcription was carried out using PrimeScript RT Reagent Kit with gDNA Eraser (Takara, Japan). The qPCR was performed with a TB Green Premix Ex Taq kit (Takara, Japan) by the manual. The qPCR program was carried out in a qTOWER3/G PCR system (Analytik Jena, Germany) with the following protocol: 95 °C for 30 s, followed by 40 cycles of 95 °C for 5 s and 60 °C for 30 s. The 16S housekeeping gene was used as the internal reference constructs. For sequences see Sup. Table 2.

**Statistical analysis** Means were calculated from five to eight independent experiments, and error bars represent standard deviation (SD). Statistically significant differences were determined using a one‐way ANOVA with Dunnett's post‐hoc test towards control and multiple sample groups comparison. Student's t‐tests were performed for the L‐II/C‐II, L−K/C−K, C−K/d‐k, dK/tri‐K, and C‐II/d‐II. All analyses were performed using GraphPad Prism 10 (GraphPad Software, Inc). Values of P < 0.05 were considered statistically significant and denoted as follows: *P<0.05, **P<0.01.

## Conflict of Interests

The authors declare no conflict of interest.

1

## Supporting information

As a service to our authors and readers, this journal provides supporting information supplied by the authors. Such materials are peer reviewed and may be re‐organized for online delivery, but are not copy‐edited or typeset. Technical support issues arising from supporting information (other than missing files) should be addressed to the authors.

Supporting Information

## Data Availability

The data that support the findings of this study are available from the corresponding author upon reasonable request.
