# Supplementary material for: Cyclization of Short Peptides Designed from Late Embryogenesis Abundant Protein to Improve Stability and Functionality
Source: Chembiochem. 2025 Feb 20;26(8):e202401013. doi: 10.1002/cbic.202401013 (PMC12007072; doi:10.1002/cbic.202401013)
Supplement: Supplementary file 1 — Supporting Information [file CBIC-26-e202401013-s001.pdf]

ChemBioChem

Supporting Information

Cyclization of Short Peptides Designed from Late Embryogenesis Abundant Protein to Improve Stability and Functionality

Yinghan Wu and Shinya Ikeno*

Supporting Information

Cyclization of short peptides designed from late embryogenesis abundant protein to improve stability and functionality

Yinghan Wu and Shinya Ikeno *

Department of Biological Functions Engineering
Kyushu Institute of Technology
Kitakyushu Science and Research Park, Kitakyushu, Fukuoka, Japan

Table S1. Primer sequences used for cloning

Primer	Sequence (5’→3’ orientation)
G6K	Fw: GAAAGACAAACTGAAAGAGAAAGCTGG
	Rev: CTCTTTCAGTTTGTCTTTCGCATCCAT
G12K	Fw: GAAAGCTAAAGAAGCATGCTTGTCGTAT
	Rev: TGCTTCTTTAGCTTTCTCTTTCAGTTTG
Nco I-LEA K-Hind III	Fw: CATGGATGCGAAAGACAAACTGAAAGAGAAAGCAAAAGAATAA
	Rev: AGCTTTATTCTTTTGCTTTCTCTTTCAGTTTGTCTTTCGCATC
Nco I-LEA II-Hind III	Fw: CATGGATGCGAAAGACGGTCTGAAAGAGAAAGCAGGTGAATAA
	Rev: AGCTTTATTCACCTGCTTTCTCTTTCAGACCGTCTTTCGCATC
Infusion dK	Fw: TTGTCGTATGACACCGAAATCCTGACC
	Rev: TTGTCGTATGACACCGAAATCCTGACC
Infusion dII	Fw: ATCCATACAATTCGACGCAATGAATCCGTT
	Rev: GGTGAAGGTACCGGCAGCATGGAT
Insert K-1.	Fw: GAAAGACAAACTGAAAGAGAAAGCTAAAGAAGGTACC GGCAGCATGGATGCG
	Rev: CGCATCCATGCTGCCGGTACCTTCTTTAGCTTTCTCTTT CAGTTTGTCTTTC
Insert K-2	Fw: GGCAGCATGGATGCGAAAGACAAACTGAAAGAGAAAGC TAAAGAAGCATGCTTGTCGTATGACACC
	Rev: GGTGTCATACGACAAGCATGCTTCTTTAGCTTTCTCTTTC AGTTTGTCTTTCGCATCCATGCTGCC
Insert II-1	Fw: AATTGTATGGATGCGAAAGACGGTCTGAAAGAGAAA GCTGGTGAAGGTACCGGC
	Rev: GCCGGTACCTTCACCAGCTTTCTCTTTCAGACCGTCT TTCGCATCCATACAATT
Insert II-2	Fw: AGCATGGATGCGAAAGACGGTCTGAAAGAGAAAGCTG GTGAAGCATGCTTGTCGT
	Rev: ACGACAAGCATGCTTCACCAGCTTTCTCTTTCAGACC GTCTTTCGCATCCATGCT
Tri-K	Fw: CGATGCGAAAGACAAACTGAAAGAGAAA GCTAAAGAAGGTAC
	Rev: CTTCTTTAGCTTTCTCTTTCAGTTTGTCTTTCGCATCGGTAC

Table S2. Primer sequences used for qPCR

Primer	Sequence (5’→3’ orientation)
SICLOPPS-qPCR	Fw: TGGGTGTCTGATTCGTGCCA
	Rev: CGTCATTTGCCGCAGTACCA
pBAD-LEAK-qPCR	Fw: TGGATGCGAAAGACAAACTGAA
	Rev: TGAGATGAGTTTTTGTTCGGGC
pBAD-LEA II-qPCR	Fw: GGATGCGAAAGACGGTCTGA
	Rev: TGAGATGAGTTTTTGTTCGGGC
16S	Fw: TGGATCAGAATGCCACGG
	Rev: ACCTTGTTACGACTTCACC
